
JOURNAL INFORMATION
==============================
NLM Title Abbreviation: J Child Orthop
Journal ID: J Child Orthop
NLM Journal ID: 101313582

Journal of Children's Orthopaedics

ISSN: 1863-2521
EISSN: 1863-2548
Publisher: SAGE Publications

ARTICLE INFORMATION
==============================
PMCID: PMC3955427
DOI: 10.1007/s11832-014-0579-y
Article ID: 579
Article version: 1
Subjects: Author Index

Author Index for Supplement 1, 2014, Belgium

Electronic publication date: 2014 Mar 15
Print publication date: 2014 Apr
Volume: 8
Issue: Suppl 1
First page: 1
Last page: 5
Copyright: © The Author(s) 2014
License: This article is distributed under the terms of the Creative Commons Attribution License which permits any use, distribution, and reproduction in any medium, provided the original author(s) and the source are credited.
License URL: https://creativecommons.org/licenses/by/4.0/

issue-copyright-statement: © The Author(s) 2014

==============================

Abdelaziz, Tarek EP89
Abelin-Genevois, Kariman OP45
Abood, Ahmed EP2
Accadbled, F OP25, OP73
Adetchessi, Tarek PP7
Agus, Haluk EP22
Ahmad, Alaaeldin EP63
Aiona, Michael OP64
Aird, James EP52
Akbarnia, Behrooz PP10
Alfaro, Maria Dolores OP5
Ali, Farhan EP34
Allington, Nanni EP21
Alshameeri, Zeiad PP18
Aminian, Kamiar OP65
Andras, Lindsay OP7
Andreacchio, Antonio EP76
Anticevic, Darko EP30
Antonio, Fontanarosa EP25
Antonioli, Diego PP23
Armand, Stephane EP7
Ashby, Elizabeth EP82, OP12
Aslan, Madeleine EP85
Asokan, Ajay EP19, OP22
Atef, Ashraf EP13
Audouin, C OP73
AuréGan, Jean-Charles EP65
Authier, Guillaume OP33

B. R. De Mattos, Camila OP64
Bache, Ed OP28
Bachy, Manon PP19
Baindurashvili, Alexey EP14
Baker, Markus EP82, OP12
Barbier, Dominique EP1, OP13
Barrett, Kody OP7
Barrios, Carlos OP18
Battibugli, Simone EP23
Beaty, James EP75, OP38, OP39, PP15, PP16
Bekmez, Senol PP13
Belthur, Mohan EP34
Berger, Leopold OP15
Bergerault, Francois PP9
Bergerault, François EP69
Bergovec, Marko EP30
Bintcliffe, Fiona EP52, OP31
Birke, Mr Oliver OP29
Black, Sheena OP40
Blanco, John EP66
Blondel, Benjamin OP11, OP42, OP49, PP7
Bocahut, Nicolas EP12
Bollini, Gerard OP11, OP42, OP48
Bongiovanin, David PP14
Bonnard, Christian EP69
Bonnard, Christian PP9
Boulay, Christophe EP10
Bowen, Lowri EP52
Boye, Gloria OP20
Bozoglan, Muhammed EP22
Braga Motta, Giovanna EP23
Bregou Bourgeois, Aline OP65
Brink, Rob OP52
Bruce, Colin EP36, OP76
Brun, Myriam EP3
Buckingham, Rachel OP31
Bukharev, Edgar EP49
Bulstra, Sjoerd EP87
Burgos-Flores, Jesus OP18
Burian, Michal EP4
Burnei, Gheorghe EP50, EP77
Burns, Joshua EP28
Buyukdogan, Kadir OP54

Cabrera GonzáLez, Marisa PP21
Cage, Jason OP40, PP8
Cambon-Binder, Adeline OP3
Campagnolo, João PP3
Canavese, Federico EP76, OP77
Cannavò, Luca EP18
Carry, Patrick PP4
Carvalho, Miguel PP3
Cassiano Neves, Manuel PP17
Castanier, Elisabeth EP10, OP33
Castelein, René EP64, OP47, OP46, OP52, PP12
Castellani, Christoph OP15
Cavaignac, E OP25
Cekanauskas, Emilis EP78
Celarek, Anna OP15
Celilov, Rustem PP13
Céline, Klein EP25
Ceroni, Dimitri EP1
Cervellione, Raimondo EP34
Cervera, Javier OP18
Chahine, Ali Khalil EP78
Chang, Chia OP4
Chatzipantelis, Stefanos PP6
Chaumoitre, Karine PP7
Chen, Mei OP4
Cheng, Jack EP64, OP52
Cheon, Jung-Eun OP26
Cho, Tae-Joon EP45, EP56, OP26
Choi, Eun Seok OP26
Choi, Eun-Seok EP45
Choi, In Ho EP45, EP56, OP26
Chomiak, Jiri EP54
Chomiak, Jirí EP4
Chomicki, Mariusz EP71
Chong, David EP33, EP37, OP30
Choufani, Elie OP11, OP42
Christophe, Glorion EP25
Chu, Winnie EP64, OP52
Chua, Joyce EP59
Chung, Chin Youb EP45, EP56, OP26, OP35, OP66, PP22
Church, Chris OP60, PP2
Clarke, Prof Nicholas OP23
Clements, Nick OP74
Clohisy, John OP32
Coggings, Di EP26

Colo, Dino PP12
Cooper, George OP28
Cooperman, Daniel EP31, EP32, EP40, OP8, PP1, EP55, OP71
Cordasco, Frank EP70
Covaro, Augusto EP62, PP14
Cravino, Mattia EP76, PP23
Cuomo, Anna OP1
Czop, Andrzej EP71

Dare, David EP66
Davidson, Austin EP61
De Bodman, Charlotte PP9
De Coulon, Geraldo EP7
De Courtivron, Benoit EP69
De Courtivron, Benoit PP9
De Gauzy, J OP25, OP73
De Gheldere, Antoine OP31
De Rosa, Vincenzo EP76
Debiparshad, Kevin EP33, EP37, OP30
Dede, Ozgur PP13
Degliute-Muller, Ramune EP78
Deignan, Brian EP57
Del-Pozo, Eduardo EP3
Demirkiran, Gokhan PP13
Desai, Ronak OP8
Di Gennaro, Giovanni Luigi PP23
Dimitriadis, Dimitris PP6
Do, Patrick (Khanh) OP64
Dodwell, Emily EP66, OP41
Domos, Gyula OP34
Donzelli, Onofrio PP23
Dormans, John PP12
Drummond, Denis PP12
Duart, Julio EP3
Dubousset, Jean EP65
Dungl, Pavel EP4, EP54
Dussa, Chakravarthy EP88

Easton, Victoria EP15
Eastwood, Deborah EP82, OP12, OP28
Edobor-Osula, Flo OP41
Edwards, Tomos EP52
Eichler, Johannes OP15, OP16
Eidelman, Mark EP44, EP48, OP58
Elkhosousy, Mohamed PP11
El-Sayyed, Adel EP89
Emans, John PP10
Estivalezes, Erik OP45
Ey Batlle, Anna EP62, PP14
Farhang, Kathleen OP8, PP1
Fay, Renaud OP17
Feng, Jing OP64
Fernandez, Ana OP5
Ferrero, Emmanuelle OP49
Finidori, Georges EP27
Fischerauer, Stefan OP15, OP16
Fitoussi, Franck PP19
Flinck, Marianne OP27
Flipsen, Mark OP78
Floh, Ulrike OP10
Flynn, Jack OP7
Forman, Jason EP3
Fournier, Joseph PP9
Fournier, Joseph EP69
Fraley, Sara EP32
Freychet, Benjamin EP69
Frias, Ana OP5
Frydrychova, Monika EP4, EP54
Fujii, Toshio EP9
Futami, Tohru EP43

Galban, Miguel EP46, PP5
Gallego, Maria De Los Angeles OP5
Ganger, Rudolf OP19
Garcia- Mata, Serafin EP3
Garg, Sumeet OP6
Gaston, Mark OP74
Gaumetou, Elodie EP29
Gavaret, Martine OP48
Gavriliu, Stefan EP50, EP77
Gaynor, Joseph OP63
Gdalevitch, Marie EP28
Geertzen, Joannes EP87
Gelfer, Yael OP22
George, Harvey L OP76
George, Darrell EP58
Georgescu, Ileana EP50, EP77
Gheen, W. OP40
Ghita, Raluca EP50, EP77
Gibbons, Mr Paul OP29
Gibbons, Paul EP28
Gilmore, Allison EP58
Gintautiene, Jolita EP78
Givon, Uri OP36
Gjessing, Sofie EP5
Glorion, Christophe EP27
Goldman, Vladimir EP83
Golicki, Dominik EP17
Goodbody, Christine OP7
Graham, H OP67
Grant, Richard EP40
Green, Daniel EP66, EP67, EP70
Greene, Janelle EP55, OP71
Grill, Franz EP47
Grosz, Gabor OP34
Gruzauskas, Mindaugas EP78
Gsell, Thibault OP48
Gudipati, Suri OP2

Hagglund, Gunnar OP62
Hahne, Julia OP19
Ham, John OP78, PP20
Hamada, Masayuki EP79
Hanf-Osetek, Dorota OP70
Harcke, H EP20
Hardesty, Christina EP60, PP10
Hashavya, Saar OP37
Helenius, Ilkka OP50
Hellfritzsch, Michel EP35
Henley, John OP60, PP2
Henry, Havalee EP40
Hepping, Ann EP87
Herman, Amir OP36
Hermanson, Maria OP62
Hevia Sierra, Eduardo OP18
Hidalgo-Grass, Carlos OP37
Hijazi, Abdussalam OP69
Ho, Christine OP6
Hogue, Grant EP74
Holbeck-Brendel, Jesper EP35
Hollman, Freek EP38
Holmes, Laurence EP20
Holmes Jr., Laurens EP86
Honmoto, Paulo EP23
Horn, Joachim OP55
Horvath, Nikoletta OP34
Hosny, Gamal OP56
Howard, Andrew OP1, OP61
Huber, Hanspeter OP17
Huguet, Sandrine EP85
Humphries, Paul EP19, OP22
Hutchinson, Rachael EP15

Ibrahim, Talal OP69
Ilharreborde, Brice EP12, EP29, OP3, OP44, OP49
Inan, Muharrem EP8
Iniguez, X OP73
Izrael, Dan EP83
Jalanko, Tuomas OP50
James, Mr Kyle OP29
Janarv, Per-Mats OP27
Jang, Hyung Gyu EP39
Janssen, Michiel OP46
Japie, Ecaterina EP50, EP77
Jasinski, Pawel OP70

Javid, Mahzad OP59
Jehanno, Pascal OP3
Jeroense, Kimberly OP75
Jimenez, Carmelo OP5
Jo, Ah Reum EP84
Jochymek, Jiri EP80
Jones, Kristofer EP70
Jones, Tamekia OP39
Joor, Fleur OP75
Jordan, Robert EP73
Journeau, Pierre EP1, EP85, OP13, OP17
Jouve, Jean-Luc EP10, OP11, OP33, OP42, OP48, OP49, PP7
Jr., J EP20
Julieanne, Sees PP2
Jung, Sung Taek EP84

Kao, Hsuan-Kai Kao EP68
Kao, Hsiun OP4
Karahan, Sevilay OP54
Karner, Christoph EP47
Kataoka, Toshiyuki EP79
Katsman, Alexander EP44
Katzman, Alexander EP48, OP58
Kawabata, Hidehiko EP42
Keith, Angela OP32
Kelly, Derek EP61, EP75, OP38, OP39, PP15, PP16
Kenis, Vladimir EP14, OP9
Khan, Ahmad OP77
Khan, Tahir EP34
Khen, Amnon EP83
Kim, Dong Wan EP53
Kim, Hyun Woo EP56
Kim, Oh-Hwa EP56
Kim, Young-Jo OP20, PP4
Kiss, Laszlo OP34
Kiss, Sandor OP34
Knox, Jeffrey PP8
Kononovich, Natalia EP1, OP13
Korpisah, Joseph EP38
Kotb, Hatem OP68
Kotlyarsky, Pavel EP44
Kranzl, Andreas OP19
Kraus, Tanja OP10, OP16
Kubat, Ozren EP30
Kubota, Hideaki EP9
Kuriyama, Kohji EP79
Kwon, Dae Gyu OP35, OP66, PP22
Kwon, Soon-Sun OP35, OP66, PP22

L. George, Harvey EP36
Laine, Timo OP51
LAKHAL, Walid PP9
Lam, Derrick EP59
Lam, Tsz EP64, OP52
Lamdan, Ron EP83, OP37
Lanca, Nuno PP3
Larue, Madeleine EP58
Lascombes, Pierre EP1, EP7, OP17
Lebel, David Eduard OP43
Lee, In Hyeok OP35, OP66, PP22
Lee, Joel EP59
Lee, Kyoung Min OP35, OP66, PP22
Lee, Sang Hyeong OP35, OP66, PP22
Lee, Seung Yeol OP35, PP22
Lee, Si Wook EP39, EP53
Lee, York Tien EP59
Lennon, Nancy OP60
Li, Wei OP4
Liberman, Boaz OP36
Lim, Kevin EP59
Link, Corrine EP57
Little, Prof. David OP29
Liu, Raymond EP31, EP32, EP40, EP58, OP8, PP1, EP55, OP71
Lochab, Jasjit OP61
Loeffler, Joerg OP16
Lopez, Tamara OP5
LORET, Jean Edouard PP9
Lourenco, Alexandre EP23
Lund, Teija OP51
Lyman, Stephen EP66, OP41

Mackenzie, William EP57
Mader, Konrad OP78, PP20
Mahdavi, Saboura OP22
Maheshwari, Raj EP36
Mainard-Simard, Laurence OP17
Maizen, Claudia EP26
Malaga-Shaw, Olivia OP22
Marangoz, Salih OP54
Marengo, Lorenza EP76
Mariani, Benoit OP65
Marks, David PP10
Martinelli, Alessandro PP23
Martinelli, Elisabeth OP16
Marx, Robert EP67
Mary, Pierre PP19
Mattei, Jean-Camille EP10
Mattila, Mikko OP50
Maximin, Marie-Christine OP48
Mazda, Keyvan EP12, EP29, OP3, OP44, OP49
Mccarthy, Moira EP70
Mcdonald-Mcginn, Donna PP12
Medrano, Eduardo OP5
Merckaert, Sophie EP16
Meyer, Jean-Baptiste OP17
Michael, Bober PP11
Miladi, Lotfi EP65
Miller, Freeman OP60, PP2
Miller, Patricia OP20
Miller, Mark OP32
Millis, Michael OP20
Mindler, Gabriel OP19
Moeller-Madsen, Bjarne EP35, EP5
Moisan, Alice OP7, PP15, EP75, OP38, PP16
Møller-Madsen, Bjarne EP2, OP14
Monsell, Fergal EP52
Moon, Jae Young EP84
Morakis, Emmanouil EP34
Morin, Christian EP24
Morris, William EP31, EP32, EP40
Muchow, Ryan OP6
Muller, Florence EP85
Murphy, Ryan PP16
Murphy, Robert EP61

Naranje, Sameer OP39
Narayanan, Unni OP1, OP61
Nawabi, Danyal EP70
Neganova, Alina EP66
Neves, Manuel PP3
Newman, Christopher OP65
Ng, Bobby EP64
Nguyen, Cynthia EP58, EP55, OP71
Nielsen, Ole B. EP5
Nielsen, Peter EP35
Niiler, Tim OP60
Novacheck, Tom OP61
Novais, Eduardo PP4
Nuñez, Maria Isabel OP5

Odent, Thierry EP65
Oketani, Yutaka EP9
O'Malley, Natasha EP60
Omar, Rumana OP22
Omeroglu, Hakan EP6
Onder, Kalenderer EP22
Oner, F. OP46
Onvural, Burak EP22
OštáDal, Martin EP54
Osterman, Heikki OP51
Ovadia, Dror OP43
Ovidio, Joana PP3

Pailhé, R. OP25
Paley, Dror EP33, EP37, OP30
Pan, Ting EP66
Pan, Ting-Jung OP41
Pargas, Carlos PP5
Park, Kwang-Won EP41, EP81, OP24, OP53
Park, Moon Seok EP45, OP26, OP35, OP66, PP22
Pattison, Giles EP73
Pavone, Vito EP18
Pawelek, Jeff PP10
Pedersen, Line EP35
Peltier, Emilie OP11, OP42, PP7
Penneçot, Georges EP29
Pereira, Bruno OP77
Pérez López, Laura PP21
Pernus, Franjo OP46
Pesenti, Sebastien OP11, OP42, PP7
Philippe, Wicart EP25
Pierce, Rosemary OP64
Ploegmakers, Joris EP87
Poe-Kochert, Connie EP60, PP10
Polirsztok, Eva OP48
Popkov, Arnold EP1
Popkov, Dmitry EP1, OP13
Pothrat, Claude OP33
Potocki, Kristina EP30
Potter, Hollis EP70
Poussa, Mikko OP51
Pretell-Mazzini, Juan EP75
Promod, Prakash OP72
Protopapa, Evangelia EP19, OP22
Pruszczynski, Blazej EP20, EP86, PP2
Pullinger, Michael EP15

Radler, Christof OP19
Rahbek, Ole EP2, EP35, EP5, OP14
Ramachandran, Manoj EP26, OP31
Ramachandran, Mr Manoj OP29
Ramaesh, Rishikesan OP74
Ramanoudjame, Mira EP27
Ramavath, Ashok OP76
Ramos, Susana PP17
Rao, Guillaume OP33
Raphaël, Seringe EP25
Rawo, Tomasz EP71
Rehm, Andreas OP72, PP18
Rennie, Louise OP74
Riad, Jacques OP27, OP62
Riaz, Mohammed OP69
Riccio, Anthony EP74, OP40, OP6, PP8
Riccioli, Maria EP18
Rogers, Kenneth OP47, PP11
Rogers, Keneth PP2
Rogers, Kenneth EP57, EP86
Rohon, Marie-Ange EP10
Rompa, Paul EP38
Roposch, Andreas EP19, OP22
Rose, Ryan EP74
Roth, Sandor EP72
Rouissi, Jihane OP44
Rush, Jeremy PP15
Rutz, Erich OP67

Sagawa, Yoshimasa EP7
Sales De Gauzy, Jerome OP45
Samba, Antoine EP76, OP77
Sanghrajka, Anish EP15
Sanpera Jr, Ignacio OP18
Santana, Adolfredo EP46, PP5
Sant'Anna, Francisco PP17
Sarikaya, Ilker EP8
Sathyamoorthy, P OP76
Sawyer, Jeffrey EP61, EP75, OP38, OP39, OP7, PP15, PP16
Schiedel, Frank OP57
Schindler, Amos OP36
Schlenzka, Dietrich OP51
Schlosser, Tom EP64, OP46, OP47, OP52
Schoenecker, Perry OP32
Scholtes, Vanessa OP78
Schrouff, Isabelle EP21
Schwartz, Joshua OP60
Scott, Brian OP2
Sees, Julieanne OP60
Segui-Gomez, Maria EP3
Seo, Sang Gyo EP56
Sessa, Giuseppe EP18
Shah, Suken OP47
Shahcheraghi, Gholam OP59
Shannon, Claire PP1, PP10
Shifflet, Grant EP67
Shiguetomi-Medina, Juan EP2, OP14
Shim, Jong Sup EP56
Shin, Michelle OP61
Shutov, Roman OP13
Sigal, Amit EP48
Simanovsky, Naum EP83, OP37
Simonaityte, Rasa EP78
Šimunovic, Marko EP30
Sinha, Ankur EP36
Sitoula, Prakash PP11
Skaggs, David OP7
Sleeboom, Christien OP75
Smith, Richard EP15
Snela, Slawomir OP70
Sokól, Grzegorz OP70
Song, Hae-Ryong EP41, EP56, EP81, OP24, OP53
Song, Kwang Soon EP39, EP53
Song, Mi Hyun EP45
Son-Hing, Jochen EP60
Souchet, Philippe EP12
Southorn, Thomas EP15
Southorn, Tom OP28
Spence, David EP61, EP75, OP38, PP15, PP16
Staal, Heleen EP38
Steen, Harald OP55
Steinwender, Gehardt OP10
Stepanova, Julia EP14
Stepensky, Polina EP83
Stephens, Byron OP38
Stevens, Martin EP87
Stillwell, J. OP76
Stitzman, Melisa EP62
Stødkilde-Jørgensen, Hans EP2
Stohr, Kuldeep PP18
Straka, Miroslav EP80
Streit, Jonathan EP40
Sung, Ki Hyuk OP66
Sussman, Michael OP64
Švehlík, Martin OP10
Swider, Pascal OP45
Symeonidis, Ioannis EP3
Szoke, George OP34

Taketa, Mayuki EP9
Tamura, Daisuke EP42
Tavares, Delfin PP17
Taylor, Daveda OP60
Terebessy, Tamas OP34
Terjesen, Terje OP21
Testa, Gianluca EP18
Thacker, Mihir EP57
Thevenin-Lemoine, Camille PP19
Thomas, Simon OP31
Thomason, Pam OP67
Thompson, Norfleet PP16, PP15
Thompson, George EP60, PP10
Thorne, Rebekah EP36
Thuesing, Monika PP17

Tiruveedhula, Madhu OP2, OP23
Tleyjeh, Imad OP69
Topouchian, Vicken EP27
Trams, Marek EP71
Tribout, Laurent OP3
Truong, Walter OP61
Tschegg, Elmar OP15
Turkeltaub, Paul OP37
TvrdíK, Daniel EP4
Ty, Jennifer EP86

Uesugi, Ayako EP79
Uggowitzer, Peter OP16
Unnikrishnan, P. Nithin OP76
Urbasek, Karel EP11

Valenti, Philippe OP3
Van Der Zwan, Arnard PP20
Van Rhijn, Lodewijk EP38
Van Stralen, Marijn EP64, OP52
Ventura Gomez, Norberto PP14
Ventura GóMez, Norberto EP62
Vialle, Raphael PP19
Viehweger, Elke EP10, OP33
Viergever, Max OP46
Vilalta Vidal, Imma EP62, PP14
Villanueva, Roceli EP46
Vincken, Koen OP46, EP64, OP47, OP52
Vlachou, Maria PP6
Vlad, Costel EP50, EP77
Von Heideken, Johan OP27
Vrtovec, Tomaz OP46

Wada, Akifusa EP9
Wagner, Philippe OP62
Warner, William PP15, EP61, EP75, OP38, PP16
Wasilewski, Piotr EP17
Watz, Veronica OP27
Weinberg, Annelie OP15
Weiss, Barbara OP15
Westacott, Daniel EP73
Wicart, Philippe EP24, EP65
Widmann, Roger EP66, OP41, EP67
Wientroub, Shlomo OP43
Wilber, John OP8
William, Mackenzie PP11
Willis, Katie EP28
Wimberly, Robert EP74, OP40, OP6, PP8
Witbreuk, Melinda OP75
Wolf, Elisabeth OP10
Wright, James OP41
Wright, Jonny EP26

Yang, Hyun-Kee EP84
Yazici, Muharrem PP13
Yoo, Won Joon EP45, OP26
Yrjonen, Timo OP51

Zackai, Elaine PP12
Zaks, Ofer OP58
Zambelli, Pierre-Yves EP16, OP65
Zgoda, Marcin EP17

Open Access

This article is distributed under the terms of the Creative Commons Attribution License which permits any use, distribution, and reproduction in any medium, provided the original author(s) and the source are credited.

